# NASCITA Italian birth cohort study: a study protocol

**DOI:** 10.1186/s12887-020-1961-1

**Published:** 2020-02-19

**Authors:** Claudia Pansieri, Antonio Clavenna, Chiara Pandolfini, Michele Zanetti, Maria Grazia Calati, Daniela Miglio, Massimo Cartabia, Federica Zanetto, Maurizio Bonati

**Affiliations:** 1 Laboratory for Mother and Child Health, Department of Public Health, Istituto di Ricerche Farmacologiche Mario Negri IRCCS, Milan, Italy; 2 President Associazione Culturale Pediatri (ACP), Narbolia, Italy

**Keywords:** Clinical trial protocol [publication type], Cohort studies, Infant, newborn, Child, Infant, Health, Public health

## Abstract

**Background:**

Young children’s healthy development depends on nurturing care, which ensures health, nutrition, responsive caregiving, safety and security, and early learning. Infancy and childhood are characterized by rapid growth and development, and these two factors contribute largely to determining health status and well-being across the lifespan. Identification of modifiable risk factors and prognostic factors during the critical periods of life will contribute to the development of effective prevention and intervention strategies.

The NASCITA (*NAscere e creSCere in ITAlia*) study was created to evaluate physical, cognitive, and psychological development, health status and health resource utilization during the first six years of life in a cohort of newborns, and to evaluate potential associated factors.

**Methods:**

NASCITA is an ongoing, dynamic, prospective, population-based birth cohort study of an expected number of more than 5000 newborns who will be recruited in 22 national geographic clusters starting in 2019. It was designed to follow children from birth to school entry age for a wide range of determinants, disorders, and diseases. Recruitment of the newborns (and their parents) will take place during the first routine well-child visit, which takes place at the office of the pediatrician assigned to them by the local health unit of residence, and which is scheduled for all newborns born in Italy within the first 45 days of their life.

Data will be web-based and collected by the family pediatricians during each of the 7 standard well-child visits scheduled for all children during their first 6 years of life. Information on every contact with the enrolled children in addition to these prescheduled visits will be also recorded.

**Discussion:**

The NASCITA cohort study provides a framework in which children are followed from birth to six-years of age. NASCITA will broaden our understanding of the contribution of early-life factors to infant and child health and development. NASCITA provides opportunities to initiate new studies, also experimental ones, in parts of the cohort, and will contribute relevant information on determinants and health outcomes to policy and decision makers. Cohort details can be found on https://coortenascita.marionegri.it.

**Trial registration:**

Clinicaltrials.gov: NCT03894566.

**Ethics committee approval**: 6 February 2019, Verbale N 59.

## Background

As concisely and effectively reported in an excellent series of papers on early child development: *Childhood development is a maturational process resulting in an ordered progression of perceptual, motor, cognitive, language, socio-emotional, and self-regulation skills. The acquisition of skills throughout the life-cycle therefore builds on the foundational capacities established in early childhood.*^1^ Multiple factors influence the acquisition of competencies and skills, including health, nutrition, security and safety, responsive caregiving, and early learning. All are necessary for nurturing care [1, 2]. Nurturing care reduces the detrimental effects of disadvantage on brain structure and function which, in turn, improves children’s health, growth, and development [1, 3]. Nurturing care is characterized by a home environment that is sensitive to children’s health and nutritional needs, responsive, emotionally supportive, and developmentally stimulating and appropriate, with opportunities for play and exploration and protection from adversities [1, 4]. Nurturing care extends beyond families to include community care givers for families [1–5]. The environmental, social, economic, political, climatic, and cultural contexts can therefore affect nurturing care and early childhood development.

Infancy and childhood are characterized by rapid growth and development, and are considered critical periods of development in life that strongly contribute to health status, well-being, and behavior across the lifespan [2]. In fact, many common diseases and challenges in adult life can be traced back to early childhood [1, 6].

The heterogeneity of the population in Italy is increasing, and sociodemographic and geographic differentials (e.g., in education and migrant status) have been associated with health disparities [7, 8]. In order to adequately describe public health in Italy, epidemiological studies enrolling participants from all population groups and settings are therefore needed.

Although Italy has a public, universal healthcare system that should pose no legal or financial barriers to subgroups of the population, considerable health inequalities exist [9]. Differences arise from differences in factors such as health behavior, exposure, environment, genes, etc.

Life course approaches show that a considerable part of these inequalities is determined by exposure, health status, and development in utero and in early childhood [1, 6]. Moreover, in early childhood, children are particularly vulnerable to the influence of different factors and their interactions. While this fact is well documented, underlying mechanisms remain unclear. It is still poorly understood how specific social factors, socioeconomic status, living conditions, parental and stakeholder care, and attitudes act on the well-being of children or in creating health inequalities among children. Moreover, interactions between these factors need to be investigated [10–12].

Birth cohort studies are a powerful study design for medical and social research because they are designed to observe the impact of early exposures prospectively and at multiple time points during child development [13]. A number of birth cohort studies have been carried out [14], also in Italy [15, 16], with different aims and sizes. The overall aim of the NASCITA study (*NAscere e creSCere in ITAlia*) is to improve the understanding of the health status of Italian children early on and how it is affected by social and health determinants. Like many other cohorts, it will address multiple research questions [15, 16]. The findings will add important evidence, in terms of epidemiological data, for the development of specific prevention measures and interventions to improve the health status of children, in particular more vulnerable ones.

### Hypothesis and significance

We hypothesize that:

- differences due to environmental, sociodemographic, and parental determinants, as well as to child characteristics and physician attitudes, exist between geographic areas in a population’s health and in the use of health resources in the first few years of life;

- differences exist in the appropriateness of care provided by the National Health Service at different levels (regional, local, family pediatrician);

- differences exist between geographical settings in parental attitudes toward the recommendations concerning children’s health care and these differences may be a determinant of child development and well-being and health resource utilization;

- the existing differences between geographical settings in the opportunities for children to access educational/socialization experiences (e.g. day-care centers) may have an impact on development.

### Aims

The main aim of the NASCITA cohort is to evaluate physical, cognitive, and psychological development, and health status and health resource use during the first six years of life in a group of newborns, and to evaluate potential associated factors. The specific research questions are:

- the relationship between child development and the domains that affect nurturing care during the preschool period: health (disease prevention and treatment), nutrition (breastfeeding and dietary approach), safety and security (care and early intervention for vulnerable children), responsive caregiving (caregiving routine), and early learning (home opportunities to explore and learn);
- the association between the well-being of children and parental adherence to the recommendations for better child care and development;
- the potential factors influencing child well-being and growth and development, including the acquisition of competences;
- the differences between geographical settings in educational and socialization opportunities available for young children and in the care provided by the family pediatricians and by the National Health Service for the same needs;

## Methods

### Study area and setting

**Fig. 1:**
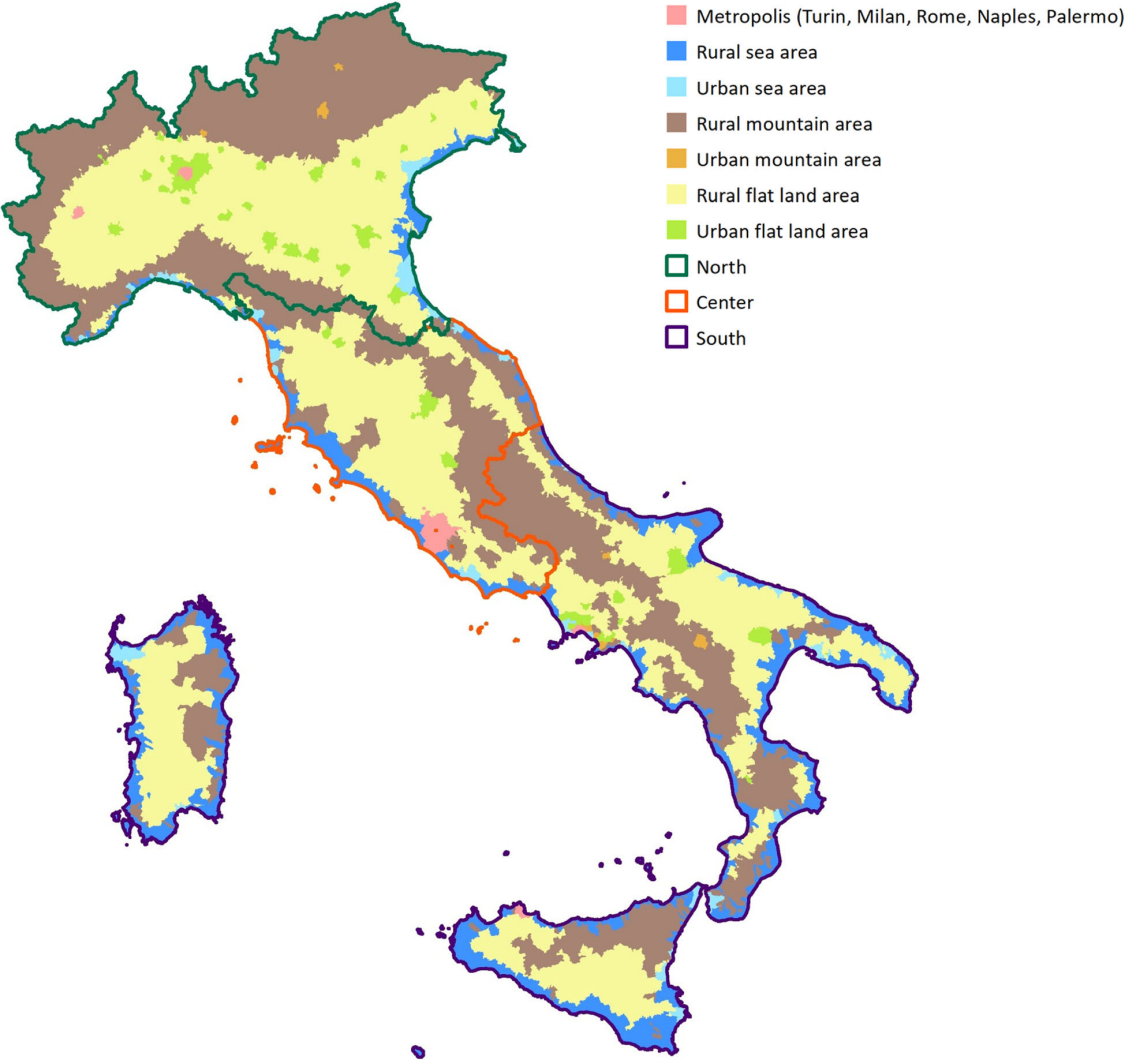
Geographical distribution of the NASCITA cohort

Italy is located in southern Europe and comprises the long, boot-shaped Italian peninsula, the southern side of Alps, the large plain of the Po Valley and some islands including Sicily and Sardinia. Almost 40% of the Italian territory is mountainous, and there is a coastline of 7600 km on the Adriatic Sea, Ionian Sea, Tyrrhenian Sea, Ligurian Sea, Sea of Sardinia and Strait of Sicily. Italy is subdivided into 20 regions and is further divided into 14 metropolitan cities and 96 provinces, which in turn are subdivided into 7960 municipalities. A gaping North–South divide is a major factor of socio-economic weakness and can be noted by the huge difference in statistical income between the northern and southern regions and municipalities [17]. Twenty-two geographic clusters were identified as representative of the country based on geographic and socio-economic characteristics and administrative divisions, using the National Statistics Institute definitions for each town/city [18]. See Fig. 1 for the geographical distribution of the NASCITA cohort. Pediatricians and newborns of all the 22 identified clusters will be involved in the study.

Italian healthcare is provided free or at a nominal charge through a network of Local Health Units (LHU). Each LHU consists of a number of healthcare districts, which are population-based territorial entities that aggregate different municipalities. Every Italian resident is registered with a family (pediatric or general) practitioner. Children are assigned to a pediatrician until they are 6 years old; afterwards, the parents can choose to register a child with a general practitioner. All adolescents > 13 years old are assigned to a general practitioner. In Italy there are about 7500 family pediatricians, for an average of 450.000 births/year [9]. About 60 newborns/year are therefore assigned to each pediatrician. All children are scheduled 7 well-child visits during their first 6 years of life (within 45 days of life and at 3, 6, 12, 24, 36, and 72 months of age). This list includes the recommended age for each well-child visit by the family pediatrician to ensure necessary preventive care, monitor a child’s growth and development, and establish a relationship between the child and his/her parents and the pediatrician.

### Study design

NASCITA is an ongoing, dynamic, prospective, population-based birth cohort study. From the start of 2019, newborns will be continuously included in the study for (at least) an entire one-year period chosen by each participating family pediatrician and will be followed prospectively until at least the age of 6 years. Given the ongoing character of the study, no maximum number of inclusions has been set. Cohort details can also be found on https://coortenascita.marionegri.it.

### Participant characteristics

The study population of the NASCITA cohort study will consist of all Italian children born during (at least) a one-year period who will be followed by participating pediatricians until the age of 6 years and whose parents agree to participate. Data on all newborns, including those with, e.g., congenital malformations, will be collected. We consider it valuable to have these data, also to be able to describe the epidemiological data more fully. The characteristics of this population will be evaluated separately in the analyses.

### Inclusion and exclusion criteria

The only inclusion criterion for participating in NASCITA is to be born in Italy during the period chosen by each pediatrician participating in the cohort study. Children whose parents do not agree to participate, or decide to withdraw, will be excluded from the study.

### Recruitment

NASCITA will be embedded in Italian pediatric primary care practice. The coordination center has collaborated over the years with a network of hundreds of family pediatricians, as documented by numerous collaborative publications [19–22], which represents the first interlocutor to whom we proposed participation in the study. The basis of this network is the national Pediatric Cultural Association (ACP), with around 2000 members consisting mainly of family pediatricians. In order to have as large a sample as possible we therefore used the already existing network to begin the first identification of the locally representative pediatricians, who were then asked to identify additional pediatricians, not necessarily belonging to the ACP, in their areas for participation. Other pediatric scientific societies and associations will be contacted to expand collaboration and the number of participating pediatricians. In this regard, detailed information concerning the study will be disseminated through national pediatric journals and internet-based resources to increase recruitment. Recruitment will be based on the voluntary participation of interested pediatricians who can guarantee seven years of professional activity so that they can follow the enrolled newborns for the whole study period. This approach to enrolling pediatricians can be defined as a mixed method using also non-probability sampling techniques (convenience and purposive sampling) applied to choose a sample of subjects/units from a population [23, 24]. In terms of the work effort requested of the pediatricians, case report forms were discussed with a group of family pediatricians and tested by them in a pilot phase. The time needed to fill the forms was recorded by each participant and difficulties or doubts were reported to the coordinating center. The participants in the pilot phase assured the coordinating team that the data collection was feasible.

Recruitment of the newborns (and their parents) will take place during the first routine well child visit scheduled for all newborns within their first 45 days of life at the office of the pediatrician assigned to them by the LHU to which they belong. Parents will receive oral and written information about the purpose and methods of the study and will be invited to participate. If they agree to participate, they will be asked to sign an informed consent. Recruitment of newborns will begin in April 2019, while recruitment of pediatricians will begin in the preceding months.

### Study population size

The NASCITA cohort is sized to have enough power to study relatively common child exposures and outcomes. Table 1 reports the national prevalence of certain health characteristics of Italian children and the expected number of cases for different enrolling scenarios in order to obtain a minimum number of participants that would permit all these characteristics to be sufficiently represented. The aim is to recruit no less than 5000, and hopefully at least 10,000, newborns with complete information collected over the period of the study. We hypothesize that, given the fact that the data collection is based on routine visits by the pediatrician, attrition in NASCITA will be irrelevant for at least the first two years. Considering the previous experience of other Italian cohorts, NINFEA [34] and PiccoliPiù [16], we estimate a 20% loss to follow-up after the first two years.

**Table 1:** National prevalence of certain health characteristics of Italian children and the expected cases for different enrolling scenarios in the NASCITA cohort study

Characteristics	Italian prevalence (%)	5.000 enrollments (n°)	10.000 enrollments (n°)	15.000 enrollments (n°)
Sex (% male) [25]	51.3	2565	5130	7695
Gestational age at birth [25]				
• < 37 weeks	6.9	345	690	1035
• 37–40 weeks	92.3	4615	9230	1385
• > 40 weeks	0.9	45	90	135
Twins/multiple birth [25]	1.7	85	170	255
Live birth rate after medically assisted procreation [25]	2.14	107	214	321
Birth defects [26]	2.0	100	200	300
Language disorders Age 3.5 years [27]	7.5	375	750	1125
Maternal smoking during pregnancy [28]	7	350	700	1050
Breastfeeding (exclusive) [29]				
• Until age 6 months	42.7	2135	4270	6405
• 6–12 months	5.5	275	550	825
Parental history of atopy [30]	22–37	1100–1850	2200–3700	3300–5550
Eczema prevalence Age 36 months-5 years [30]	21.0	1050	2100	3150
Wheezing prevalence Age 36 months-5 years [31, 32]	25.2	1260	2520	3780
Overweight prevalence Age 2–6 years [33]	16	800	1600	2400

With an expected minimum of 5000 newborns, representing about 1% of the newborns in Italy, and with an estimated 20% loss to follow-up, the resulting sample size of 4000 children will still give NASCITA enough power to study common childhood exposures and outcomes [16].

With the parents’ consent, data on children withdrawing after 12 months of age will be considered in the analysis for the relevant time period of participation (e.g. rate of exclusive breastfeeding, reading out loud, SIDS prevention, etc.). Data will be deleted upon parents’ request.

### Training and tutorial activities

Before the start of the study, family pediatricians will be involved in training activities. Local coordinators will be trained by the research team, and will be responsible for the training of their peers at the local level. A case report form (CRF) was created in an online form with the contribution of local representatives of family pediatricians and scientific committee participants. During an initial phase, a group of family pediatricians tested the electronic CRF (eCRF), leading to improvements and adding the necessary questions to achieve an eCRF that would allow a more complete and simple collection of data. The eCRF will be available online before the start of the enrollment period in order to let participating family pediatricians familiarize with the information that needs to be collected.

Central and local monitoring of the study will be scheduled with the aim to guarantee follow-up of the infants and the quality of data collected.

### Data collection

**Fig. 2:**
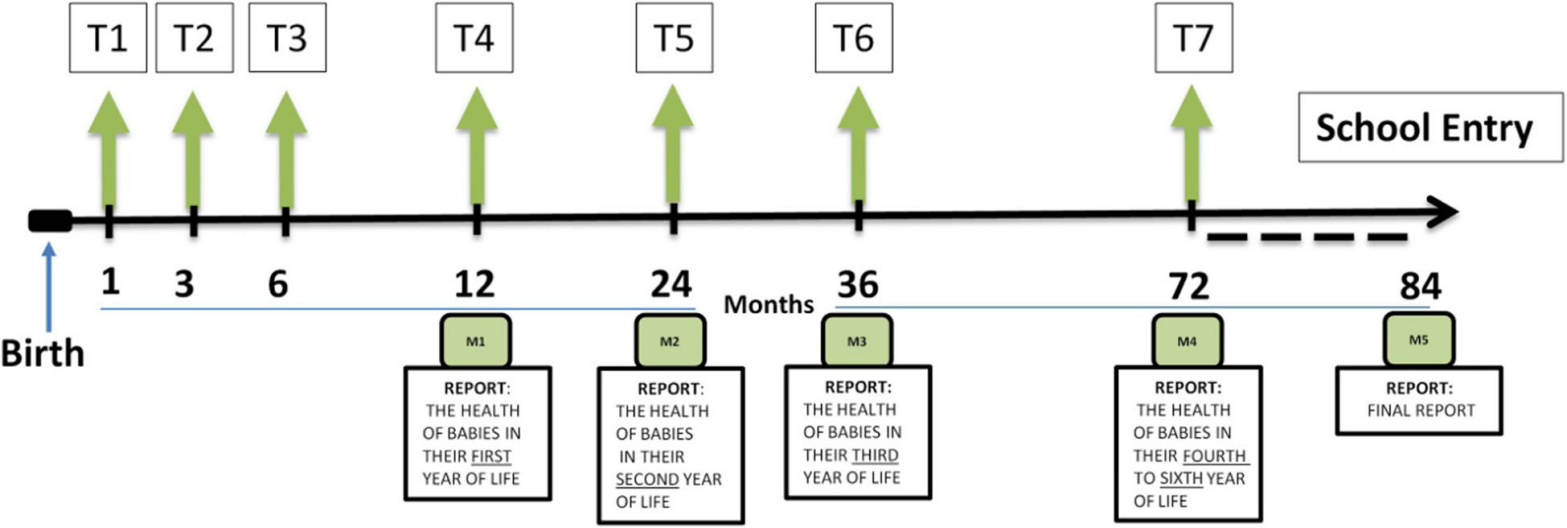
Timeline of data collection, follow-up, and milestones in the NASCITA Study

Data considered for the basic CRF are part of those routinely collected by the family pediatricians at the 7 standard well-child care visits scheduled for all children during their first 6 years of life, and data collected during each contact with the enrolled children. See Fig. 2 for the timeline of data collection, follow-up, and milestones in the NASCITA Study. In addition to the basic data, questions were added to allow the project to address specific areas such as nutrition, environment, and nurturing care. The eCRFs were consequently structured in a way that will permit us to expand data collection and analysis in these areas in a second phase. More specifically, in the future, distinct protocols will be created for potential sub-studies in these areas, also taking into consideration the research interests and expertise of the participating pediatricians in their design and in the identification of all useful data items. In order to enhance the quality of the data, the eCRF includes consistency and range checks to prevent internal inconsistencies, although the continuing review of collected data is guaranteed by the coordinating center and, in case of inconsistencies, pediatricians will be contacted. The administrators of the website (the coordinating center) will be able to view the completed forms also in a graphic format that will be updated periodically.

### Baseline data

At the first visit (within 45 days of life of the newborn), parents will be asked about parental medical history, characteristics and lifestyle, indoor and outdoor environment, and circumstances during pregnancy and around birth. Pregnancy and perinatal data will be collected also through hospital discharge documents following delivery.

### Follow-up and outcomes

The primary outcomes of the study are measures of health of the newborns/children from birth until (at least) the age of 6 years. Health outcomes of children aged 0–6 years will cover different fields including: physical development, mental/cognitive development, nutrition and allergies, environmental exposures, and preventable infectious diseases. Table 2 provides an overview of data sources and collection at the different follow-up stages.

**Table 2:** Overview of outcome measures collected by follow-up stage

Phase	Measurements
Baseline, T1 (within 45 days of life)	Demographic, socioeconomic, and health status of parents, details of pregnancy and perinatal routine, well-being of newborn, breast feeding/breast milk substitutes, living and social environment. Metabolic screening (notification). Anthropometric evaluation and physical examination. Immunization coverage. Infectious diseases. Dental health.
T2 (3 months of life)	Anthropometric evaluation and physical examination. Breast feeding/breast milk substitutes, child health, sleep. Hip ultrasound (notification). Vaccines (notification). Infectious diseases. Dental health.
T3 (6 months of life)	Anthropometric evaluation and physical examination. Neuromotor development assessment (Brazelton). Boel test. Breastfeeding status/Nutrition Vaccines (notification). Infectious diseases. Dental health.
T4 (1 year of life)	Child health, physical and psychological development, sleep, language development. Vaccines (notification). Breastfeeding status/Nutrition. Neurodevelopment evaluation. Infectious diseases. Dental health.
T5 (2 years of life)	Physical and cognitive development of child, child health, sleep, language development, and spoken language at home, media use. Language development. Breastfeeding status/Nutrition. Neurodevelopment evaluation. Vaccines (notification). Infectious diseases. Dental health.
T6 (3 years of life)	Anthropometric evaluation and physical examination. Child nutrition. Hearing check. Language development. Neurodevelopment evaluation. Vaccines (notification). Infectious diseases. Dental health.
T7 (4-6 years of life)	Physical and cognitive development of child, child health. Child nutrition. Neurodevelopment evaluation. Visual acuity examination. Vaccines (notification). Infectious diseases. Dental health.

### Analysis plan

The analysis plan for the NASCITA cohort entails the investigation of several exposures and outcomes in order to address numerous research questions.

The main independent variables that will be tested are:

Maternal/paternal age.

Education level.

Employment.

Marital status.

Parental health status.

Parental lifestyle habits (smoking, alcohol).

A few examples of the dependent variables are:

Vaccination in pregnancy.

Folic acid prophylaxis.

Birthweight.

Duration of breastfeeding.

Reading out loud.

Sleep position.

Overweight and obesity.

Neurocognitive development.

Prescription prevalence and appropriateness.

Nursery school attendance.

A few of these variables will be tested as both independent and dependent variables based on the kind of analyses (e.g., reading out loud will be the dependent variable while testing the influence of maternal age or educational level, but will be considered an independent variable when possible factors influencing children’s development are tested).

Examples of the NASCITA STUDY’S research questions are:

- the relationship between mothers’ age, education level, and geographic area (independent variables) and vaccination in pregnancy (Influenza and Tdap vaccines) (dependent variable);

- the association between maternal smoking in pregnancy (independent variable) and birthweight (dependent variable);

- the relationship between pregnancy, perinatal, and newborn growth characteristics and occurrence of adverse outcomes (i.e., obesity, hypertension, wheezing, eczema, hay fever, and asthma);

- the relationship between parents’ educational level (independent variable) and duration of breastfeeding (at discharge from hospital and at 3 and 6 months) (dependent variable);

- the relationship between parents’ educational level (independent variable) and children’s weight and BMI/percentage of overweight/obese status (dependent variable);

- the association between lifestyle factors and health inequalities and the trajectory of health in the preschool period;

- the association between geographical setting and nursery school attendance;

- the association between geographical setting and quality of care in terms of prevalence and appropriateness of drug prescriptions.

In all of the of the analyses, the effect of the geographic and environmental setting will be evaluated.

This type of general objective, involving numerous research questions, is similar to the goals of two Italian cohorts, the NINFEA [34] and the PiccoliPiù [16], as stated in their protocols.

Migrant newborns will be included in special subgroup analyses.

Categorical variables will be summarized using proportions and associations tested using chi-square or Fisher’s exact text, where applicable. Continuous variables will be summarized using means and standard deviations for normally distributed data, while skewed data will be summarized using medians. One-way ANOVA (F-value) will be used to test difference of means for normally distributed continuous variables and the *Mann Whitney U* test for skewed continuous variables. Statistical significance will be evaluated using a 95% confidence interval and a two-tailed *p-*value of < 0.05. Multivariate analyses will also be performed based on the study designs and outcomes to be evaluated.

### Organization framework

The coordination of the NASCITA study will be provided by the team of the Laboratory for Mother and Child Health of the Mario Negri Research Institute that integrates different expertise and competence with a long-standing experience in multicenter clinical research. The coordinating center will also carry out data collection, storage, management, and analysis. An independent scientific committee consisting of representatives of different disciplines and realities (including lay people) will monitor the development and results of the project. A network of local contacts (contact person per area) between pediatricians has been set up so that each node, representative of a setting, will act as a bridge to the coordinating center in conducting the study. An additional group of individual pediatricians has been identified to act as specialists in their area of expertise, e.g., environment, nutrition, and neurodevelopment.

### Materials produced

A specific web portal for the NASCITA cohort study was developed (https://coortenascita.marionegri.it) to collect data, through a web-based form, and to provide findings and other information during the study period, also with the use of graphics on the analyses and data collected based on a successful approach already reported by the coordinating center [19, 35]. The NASCITA project will use ad-hoc information material, the study website, and newsletters to keep in touch with study participants. Health promotion measures within the cohort will be implemented through the use of these tools, and will also be made available to the general population through publications on the website.

### Ethics and dissemination

The study was approved by the Fondazione IRCCS Istituto Neurologico Carlo Besta’s Ethics Committee (6 February 2019, Verbale n.59). A consent form for participation will be signed by the pediatricians upon their first access to the web site. A paper consent form will be signed by parents at the first visit. This form includes the consent to data collection at each contact with the pediatrician during the six-year study period (first 6 years of the child’s life). The filled in consent form will be stored by the pediatrician for ten years. Withdrawal from the study is guaranteed at any time both to pediatricians and parents. When consent is withdrawn, the child’s data collected up to that point will be kept in the analyses, but no further data will be collected. Standard procedures for the protection of confidential individual information will be applied according to national and international ethical recommendations and guidelines as well as national legal regulations. Data will be pseudonymized and all analyses will be conducted with fully anonymized data sets.

A newsletter report will periodically be sent to the pediatricians and uploaded on the web site. During the study, different tools will be used to update the participating families. Ad-hoc information material will be created and will be disseminated to families through newsletters and the website. Collected data will be periodically analyzed according to the aims of the project, and findings reported to lay people and the scientific community.

The coordinating center will provide the information, but the pediatricians will also be able to provide the families with information deriving from the cohort during their visits.When the enrolled children reach the age of 6 years, if not decided otherwise in the meantime, their personal files will no longer updated, but will be kept for another 10 years in the database.

NASCITA proposes to be a resource for the research community, so data will be available to public researchers outside the NASCITA research group upon request for collaborative research initiatives, after approval by the scientific committee.

## Discussion

### Application of study results

The information gathered by the NASCITA birth cohort study will be valuable for child health care and public health policy making. Information concerning the children will be collected at specific ages that coincide with routine contact moments, so findings from NASCITA that can be translated into parental advice or other preventive measures can directly be incorporated into routine protocols and reach a large group of children and their parents at once. Furthermore, study results on (modifiable) risk factors, disease prognosis, and medication use may also be relevant for family pediatricians.

Moreover, NASCITA findings may aid policy and decision makers, who need scientific evidence to develop and implement prevention and intervention strategies. NASCITA will progressively build on a database containing policy relevant information on a broad range of determinants and health outcomes that may be beneficial in responding to certain public health issues. NASCITA results may also contribute to the evidence towards the need to build up a permanent national observatory on child health and development.

### Strengths and limitations of this study

NASCITA is entirely embedded in the child health care practice foreseen by the National Health Service and provided by family pediatricians. Recruitment and follow-up coincide with routine contact moments, so broad participation and follow-up rates are expected. Collaboration with other cohorts is foreseen. The NASCITA cohort data will be linkable and integrable with other data sources, such as routinely collected health data or data from other projects, as part of scientific collaborations.

High participation rates would allow an appropriate description and evaluation of all the different national territorial clusters. Moreover, NASCITA will provide opportunities to initiate new, experimental studies in subgroups of the cohort, and will contribute relevant information on determinants and health outcomes to policy and decision makers.

Since loss to follow-up is always a cause for concern in cohort studies and should be minimized, efforts have been made to establish a close and trust-based relationship with all participants. These efforts involve the creation of ad-hoc information material, the website, and newsletters to keep in touch with the study participants and to apply health promotion measures within the cohort. The estimated 20% loss to follow-up would, in any case, lead to a sample size that is large enough to be able to study common childhood exposures and outcomes. With the parents’ consent, data on children withdrawing after 12 months of age will be considered in the analysis.

A limit of the NASCITA study (as with any observational study) is the possibility of selection bias in the study population. The pediatricians recruited represent a co-operative sample, not a random sample, and should not be considered to be representative of the population of family pediatricians. The pediatricians, for example, could be more sensitive to better care practices or recommendations, and could influence the parents accordingly, promoting, for example, reading out loud to children. The target population of the study, however, will be the newborns (and their families) who are assigned to the pediatricians by the LHU based on places that have been freed up with those pediatricians. No selection bias in the population should therefore occur.

The rising number of migrant patients means increasing potential language barriers in the communication between a healthcare practitioner and a patient who speaks a different language, and miscommunication may occur in healthcare settings [4]. Although this is a situation that family pediatricians have to face daily, some foreign language speaking parents, in particular recent immigrants, may decide not to participate in the study and this may create a minimal selection bias.

## Data Availability

Not applicable.

## Abbreviations

CRF: Case Report Form
eCRF: Electronic Case Report Form
LHU: Local Health Unit
NASCITA: *NAscere e creSCere in ITAlia*
